# Complexities and approaches for deriving longitudinal daily morphine milligram equivalents using electronic health record prescription data

**DOI:** 10.1093/jamiaopen/ooaf053

**Published:** 2025-06-16

**Authors:** Samantha H Chang, Shawn C Hirsch, Sonia M Thomas, Mark J Edlund, Rowena J Dolor, Timothy J Ives, Charlene M Dewey, Padma Gulur, Paul R Chelminski, Kristin R Archer, Li-Tzy Wu, Janis Curtis, Adam O Goldstein, Lauren A McCormack, INSPIRE Study Team, Alyssa Anderson, Federica B Angel, Deborah Barrett, Georgiy V Bobashev, Lynn A Bowlby, Fred Wells Brason, Audrina J Bunton, Ranee Chatterjee, Chloe R Coletta, Rogelio A Coronado, Penney Cowan, David L Crenshaw, Sofia Z Dard, Alanna DiVietro, David A Edwards, Miriam H Feliu, Parul M Goyal, Luke M Hunter, Jolene Jacquart, Julia A Jermyn, William S John, Shawn F Kane, Curtis A Kieler, Susan F Kroop, Alejandra P Madrid, Sonia Matwin, Stephanie McInnis, Lindsey C McKernan, Puneet Mishra, Amanda H Nelli, Keisha-Gaye O’Garo, Niyati S Patel, Katrice M Perry, Steven D Prakken, Amanda Priest, Vinay C Reddy, Catherine P Sanford, Emily A Smith, Linda Squiers, Claudia M Squire, Stormie G Stafford, Mark D Sullivan, Jessica Thompson, Susan B Trout, Kathy L Vu, Laura K Wagner, Jenna L Walters, Ashley M Wheeler

**Affiliations:** Center for Clinical Research, RTI International, Research Triangle Park, NC 27709, United States; Center for Clinical Research, RTI International, Research Triangle Park, NC 27709, United States; Center for Clinical Research, RTI International, Research Triangle Park, NC 27709, United States; Community Health & Implementation Research Program, RTI International, Research Triangle Park, NC 27709, United States; Department of Medicine, Duke University School of Medicine, Durham, NC 27710, United States; Department of Medicine, School of Medicine, University of North Carolina at Chapel Hill, Chapel Hill, NC 27599, United States; Division of Practice Advancement and Clinical Education, Eshelman School of Pharmacy, University of North Carolina at Chapel Hill, Chapel Hill, NC 27599, United States; Department of Medicine, Vanderbilt University Medical Center, Nashville, TN 37232, United States; Department of Anesthesiology, Duke University School of Medicine, Durham, NC 27710, United States; Department of Medicine, School of Medicine, University of North Carolina at Chapel Hill, Chapel Hill, NC 27599, United States; Department of Orthopaedic Surgery, Vanderbilt University Medical Center, Nashville, TN 37232, United States; Department of Physical Medicine and Rehabilitation, Vanderbilt University Medical Center, Nashville, TN 37232, United States; Department of Medicine, Duke University School of Medicine, Durham, NC 27710, United States; Department of Psychiatry and Behavioral Sciences, Duke University School of Medicine, Durham, NC 27710, United States; Clinical and Translational Science Institute, Duke University School of Medicine, Durham, NC 27710, United States; Department of Family Medicine, School of Medicine, University of North Carolina at Chapel Hill, Chapel Hill, NC 27599, United States; Communication Practice Area, RTI International, Research Triangle Park, NC 27709, United States

**Keywords:** opioids, morphine milligram equivalents (MME), electronic health records, prescriptions, chronic non-cancer pain

## Abstract

**Objective:**

To describe challenges and solutions for calculating longitudinal daily opioid dose in morphine milligram equivalents from electronic health record prescriptions for a clinical trial of voluntary opioid reduction in patients with chronic non-cancer pain.

**Materials and Methods:**

Researchers obtained opioid prescriptions for 525 participants from the National Patient-Centered Clinical Research Network datamart at three health systems. Daily opioid dose was calculated using dose conversions and summing across prescriptions after applying assumptions, reviewing suspect prescribing patterns, and removing spurious prescriptions.

**Results:**

Out of 16 071 extracted prescriptions, 1207 (8%) were unusable, and 14 864 (92%) were analyzed.

**Discussion:**

Numerous challenges were identified related to incomplete data, inaccurate refill dates, and overlapping or duplicate prescriptions.

**Conclusion:**

Using electronic prescription data to calculate daily doses of opioid consumption is challenging and requires significant cleaning prior to use in research. This paper recommends steps to review and clean electronic opioid prescription data.

## Background and significance

Electronic health records (EHRs) are useful data sources for research as they are comprehensive, real-time systems that contain standardized variables and vocabularies, which reduces the burden of data collection compared to traditional research methods.^1^ EHRs are particularly applicable to opioid research because most states require providers to electronically prescribe opioids.^2^

To compare opioid dosing in research, prescriptions are converted to the standard unit of morphine milligram equivalents (MME).^3^ Although the daily MME for a prescription can be derived directly within EHR systems equipped with calculators or using external calculators,^4^^,^^5^ MME may not be calculatable or accurate if data fields are missing or entered incorrectly. Moreover, methodologies for calculators can differ, and details of derivation processes are not always publicly available.^3^ While the calculation of MME for a single prescription is well-established, a literature review revealed no information on how to harmonize opioid prescription data to accurately derive longitudinal average daily MME across multiple healthcare systems where prescribing patterns and available data may differ.

The calculation of longitudinal daily MME using EHR opioid prescriptions identified challenges, including incomplete, duplicate, and overlapping prescriptions. With input and validation from prescribing physicians, the process of calculating daily MME in the context of longitudinal patterns and the cleaning methods to address prescription data complexities are described here. The goal of this article is to provide insights to help researchers clean, harmonize, and evaluate prescription opioid use over time and plan for potential challenges with electronic opioid prescription data in research studies.

## Methods

### Setting

The Integrated Services for Pain: Interventions to Reduce Pain Effectively (INSPIRE) randomized controlled trial (NCT03454555) evaluated the effectiveness of shared decision making (SDM) and motivational interviewing with cognitive behavioral therapy (MI + CBT) on voluntary opioid reduction for chronic non-cancer pain (CNCP) patients. This study enrolled 525 participants (average age of 58 years and 63% female) from 3 health systems between June 2019 and March 2022: Duke University (N = 181), University of North Carolina (UNC, N = 173), and Vanderbilt University Medical Center (N = 171). All sites use Epic, the most utilized EHR system in the United States.^6^ The study protocol with a full description of the methods was previously published.^7^

### Data sources

All sites were members of the National Patient-Centered Clinical Research Network (PCORnet) Science, Technology and Research partnership (STAR) Clinical Research Network^8–10^ and maintained an EHR datamart, standardized to the PCORnet Common Data Model (CDM), allowing for efficient use in approved multi-site research studies. This study used PCORnet CDM Version 6.1.^11^ Research Triangle Institute (RTI) International obtained prescriptions and other EHR data that were approved for sharing by participant informed consent and UNC’s Institutional Review Board by collaborating with sites to implement a data extraction program written by RTI. Sites matched participants to their medical record numbers to extract their EHR data as depicted in Figure S1. Participants also completed a web-based survey at baseline, 6 months, and 12 months to provide self-reported opioid use and dose reduction goals.

Sites’ extracted prescription data were combined and merged with the Centers for Disease Control and Prevention (CDC) MME conversion data file^12^ based on each medication’s National Drug Code. The MME conversion factor for each opioid type in this study is in Table S1. The distribution of opioids in the analytic dataset is in Table S2.

### Derivation of MME

**Figure 1.**
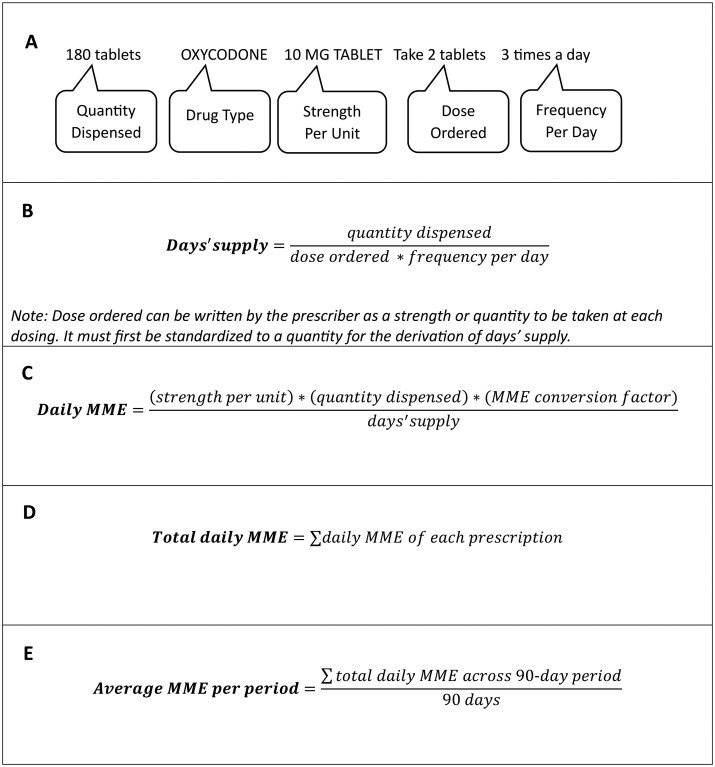
Components of a prescription [Panel A] used in the derivation methods for days’ supply for a single prescription [Panel B], daily morphine milligram equivalent (MME) for a single prescription [Panel C], total daily MME for a single participant for all opioid prescriptions in a given day [Panel D], and average MME for all opioid prescriptions over a 90-day period for a single participant [Panel E].

Each extracted prescription included the order and start dates; drug type, strength per unit, and total quantity dispensed (eg, oxycodone, 10 mg, 180 tablets); and dosing instructions of how many units to take at one time (dose) and how many times a day (frequency) (Figure 1, Panel A). Prescription end date was provided but not used in calculations because it was unreliable according to prescribing clinicians and CDM documentation.

There were also “raw” non-standardized prescription variables within the database that were used when the CDM-standardized fields were not detailed enough for MME derivation. These fields store the original Epic value prior to mapping to the CDM-standardized value (eg, raw frequency of “5 times daily” is captured as “other” in the CDM). A programmer at each institution transcribed their raw data according to CDM specifications and data implementation guidance.^11^

Change in average daily opioid dose, measured in MME, was averaged over 90-day increments at baseline (90 days before randomization), 3, 6, 9, 12, 15, and 18 months post-randomization. Average daily opioid dose was derived from the prescription data in 4 steps.^13^ First, days’ supply, or the total number of days covered by the prescription, was calculated (Figure 1, Panel B), and this quantity was used to calculate each prescription’s daily MME (Figure 1, Panel C). Sample prescriptions’ derived daily MME were validated with online calculators^4^^,^^5^ and approved by prescribing physicians. Next, medication start dates were used to assign daily MME to each study day covered by a single prescription. For multiple prescriptions taken on the same day (eg, a long-acting opioid with a short-acting opioid for breakthrough pain), all individual prescription MME were summed to derive a total daily MME for that day (Figure 1, Panel D). Finally, change in average daily dose was derived by averaging all total daily MME over 90-day increments (Figure 1, Panel E). The index date (study day 1) was defined as 90 days prior to the date of randomization. The follow-up period was fixed at 18 months post-randomization, and the full prescription data collection period lasted 21 months.

### Data cleaning

**Figure 2.**
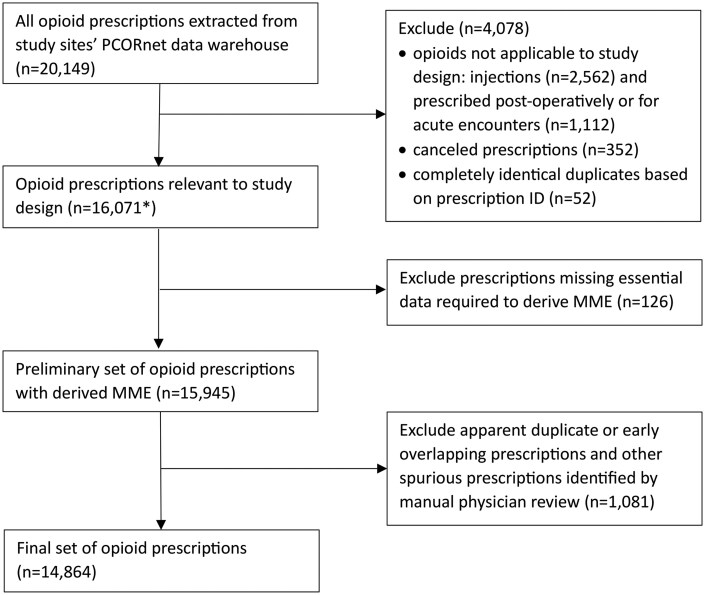
Flow diagram of exclusion steps to develop prescription analytic dataset. *The data cleaning steps described in Table 1 and Table 2 are applicable to this starting number of opioid prescriptions.

Prior to performing any data cleaning steps, prescriptions not relevant to the study design were removed (Figure 2). Most daily MME calculations could not be derived without data cleaning or applying assumptions. Additionally, temporal prescription patterns were observed that were clinically implausible, suggesting that some refills had incorrect start dates and some duplicate or canceled prescriptions were included in the data. The results section describes the cleaning procedures, which were reviewed and approved by prescribing physicians.

## Results

### Overview of prescription removals

The PCORnet datamart extraction provided 20 149 opioid prescriptions. Initially, 4078 prescriptions were excluded because they were canceled (n = 352, 1.7%), an exact duplicate based on prescription ID (n = 52, 0.3%), or medications that did not align with the study’s focus on long-term opioid therapy for CNCP (injections, administered during acute encounters, or one-time post-operative doses; n = 3674, 18.2%), leaving 16 071 opioid prescriptions cleaned according to the methods described in this paper. Of these, 1207 (7.5%) prescriptions were removed because MME could not be calculated due to incomplete data, or they were determined to be erroneous duplicates. After cleaning, 14 864 (92.5%) prescriptions remained for analysis (Figure 2).

### Calculation of MME

While calculating the MME for each prescription, several challenges were encountered related to missing frequency, quantity, or dose fields that required a series of assumptions (Table 1). An overarching assumption was made that all prescriptions were filled and that patients took them as prescribed and to completion. While impossible to know how medications were taken, all prescriptions are available to patients for use and so were included. The most common resolutions included assuming the medication was taken at the maximum frequency for pro re nata (PRN) prescriptions (59.5% of 16 071 prescriptions), assuming a 30-day supply when frequency instructions or quantity dispensed were missing (16.9%), and deriving quantity dispensed when not provided (6.1%). When the dose was a range (eg, 30–60 mL or 2–3 tablets) it was assumed that the lowest dose was taken (5.3%). It was also assumed that all EHR-indicated refills for Schedule III (eg, acetaminophen with codeine, buprenorphine) and IV (eg, tramadol) opioids, for which refills are allowed,^14^ were filled and taken accordingly (4.1%). Lastly, certain medications required special considerations to calculate MME due to their unique routes of administration or biochemical properties that affect dosing (Table 1, #8–12, #14; Table S1). Because methadone has a long and unpredictable half-life, its conversion factor is non-linear, increasing at higher doses. Solutions, typically prescribed in units of milliliters, had to be converted into milligrams to calculate MME. The conversion factor for buprenorphine patches is multiplied by the length of wear (7 days).

**Table 1.** Challenges related to deriving days’ supply and daily morphine milligram equivalent (MME) for a single opioid prescription from the electronic health record.

Challenge	Resolution	Example	**n (%)** ^a^
**Ambiguities in instructions**			
1. PRN (as needed) prescriptions	Assumed taken at maximum frequency	“Every 6 hours PRN” is assumed to be one dose every 6 hours, with no variation.	9558 (59.5%)
2. Dose written as a range	Assumed lowest dose was taken	A solution opioid with a dose of 30-60 mL is assumed to be 30 mL per dose.	847 (5.3%)
3. Refills for Schedule III and IV controlled substances	Assumed participants filled all refills with no skipped days (increased days’ supply and quantity dispensed)	A 30-day Rx of 60 tablets with 2 refills is assumed to have a days’ supply of 90 days and a quantity dispensed of 180 tablets.	661 (4.1%)
**Missing data**			
4. Frequency instructions or quantity dispensed is missing and days’ supply cannot be calculated	Assumed a 30-day supply, the most common observed period for opioids and suggested by prescribing physician	Drug A with missing frequency instructions is assumed to have a days’ supply of 30.	2708 (16.9%)
5. Quantity dispensed is missing	First assumed a 30-day supply (challenge #4), then calculated quantity using days’ supply, frequency, and dose	Drug B prescribed as a dose of 1 tablet taken 2 times daily for 30 days has an imputed quantity of 60 tablets (1 tablet per dose × 2 doses per day × 30 days).	980 (6.1%)
6. Rx is missing essential data required to calculate MME	Removed Rx from analytic dataset	If quantity dispensed and frequency are missing, then daily MME cannot be derived and the Rx is removed.	126 removed (0.8%)
7. Frequency instructions are missing for Rx with “24 HR” or “12 HR” in the medication name	Assumed to be taken per frequency in the medication name; days’ supply calculated based on frequency in medication name	60 EXTENDED RELEASE 12 HR tablets with no frequency instructions is assumed to be taken twice a day over 30 days.	54 (0.3%)
**Drug-specific considerations**			
8. Patch Rx frequency instructions are missing	Patches with “WEEKLY” in the medication name assumed to be worn for 7 days; otherwise, assumed to be worn for 3 days, as suggested by prescribing physician	BUPRENORPHINE WEEKLY TRANSDERMAL PATCH with missing frequency instructions is assumed to be worn for 7 days per patch.	133 (0.8%)
9. Methadone dose is missing	Assumed dose is equal to strength per unit, as suggested by prescribing physician	METHADONE TABLET where dosing instruction is missing is assumed to be 1 tablet per dose.	119 (0.7%)
10. Patch or buprenorphine film dose is missing	Assumed a single dose is 1 patch or film, the most common observed dose for patches and buprenorphine films	FENTANYL TRANSDERMAL PATCH without dosing information is assumed to be taken as 1 patch per dose.	112 (0.7%)
11. Patch quantity dispensed is missing	Assumed quantity dispensed (number of patches) is equal to dose; days’ supply is equal to length of wear per patch	BUPRENORPHINE WEEKLY TRANSDERMAL PATCH written as a dose of 2 patches and 7 days per patch is assumed to have a total quantity dispensed of 2 patches and a days’ supply of 7.	111 (0.7%)
12. Solution quantity dispensed is missing	First assumed a 30-day supply (challenge #4), then derived quantity using days’ supply, dose, frequency, and strength per unit (if needed to calculate quantity in mL)	A 5MG/5ML oral solution prescribed to be taken as 5 mg every 4 hours over 30 days is assumed to have a quantity dispensed of 900 mL ([30 days × 5 mg per dose × 6 times per day]/strength 1 mg/mL).	56 (0.3%)
13. Post-operative single dose Rx	Removed from calculation of daily MME in analysis as they did not contribute to chronic opioid dosing	Drug C prescribed to be taken every 30 minutes and administered during an acute visit where order date, start date, and end date are all the same was removed from analysis.	46 removed (0.3%)
14. Methadone frequency instructions are missing and milligrams of methadone per day cannot be calculated	Assumed MME conversion factor is 4, corresponding to 20 mg/day, the median value observed for all methadone Rxs where frequency is provided	METHADONE TABLET with missing frequency instructions, and therefore no derived milligrams of methadone consumed per day, is assumed to have a conversion factor of 4.	7 (<0.1%)

Quantity dispensed = total number of units to be taken over prescription duration; days’ supply = total number of days covered by the prescription duration; dose = total strength or quantity to be taken at one time; frequency = how many times to take the dose per day.

a Challenges are not mutually exclusive. A single prescription may be counted under multiple challenges. Percentages are calculated out of the 16 071 extracted opioid prescriptions relevant to the study’s design.

### Correction of start dates for refills and removal of duplicate prescriptions

A second set of data cleaning steps addressed irregularities in start dates across prescriptions and identified refills versus apparently spurious duplicate records, defined as the same medication prescribed over the same period (Table 2, #1–4).

**Table 2.** Challenges related to determining longitudinal patterns of daily morphine milligram equivalent (MME) across prescriptions from the electronic health record.

Challenge	Resolution	Example	**n (%)** ^a^
**1. Overlapping Rxs not canceled** ≥2 Rxs for same medication, dose, quantity, and start date, where ≥1 Rx had: (1) end date before start date, (2) end date before or same as order date, or (3) end date missing	Removed Rx(s) assumed to be a faulty entry that had not been canceled when no subsequent gap was indicative of an intended refill	1st Rx was removed between 2 Drug A Rxs with the same quantity, frequency, and 26NOV22 start date, where the end date of the 1st Rx is 26NOV22 and the 2nd is 21DEC22.	460 removed (2.9%)
**2. Duplicates with similar start dates** 2 Rxs for same medication, frequency, and quantity dispensed, where start date of the 2nd Rx is 1-2 days after start date of the 1st	Removed Rx with the later start date, assumed to be a duplicate entry when no subsequent gap was indicative of an intended refill	Later Rx was removed between two 30-day Rxs of Drug B with the same quantity and frequency, where the 1st Rx starts on 27AUG20 and the 2nd on 29AUG20.	520 removed (3.2%)
**3. Sequential refills** 2-3 Rxs for same medication, dose, quantity dispensed, and start date, followed by 1-2 months of no Rxs	Changed the start date of the subsequent Rxs, assuming they were intended to be filled in succession	Three 30-day Rxs of Drug C with same quantity, dose, and start date of 10JUN22 with no Rxs for 60 days after 10JUL22. Start dates for 2 Rxs were changed to 10JUL22 and 09AUG22.	320 modified start date (2.0%)
**4. Physician-reviewed duplicates** Manual physician review of Rxs for same medication, dose, quantity dispensed and start date (not captured in #3)	Per physician review of subject’s Rx patterns: 1. removed if Rx substantially overlapped with a matching Rx, or 2. added 30 days to start date if determined to be a refill	For two 30-day Rxs of Drug D with the same quantity dispensed, frequency, and 12FEB21 start date and no same medication within the length of the days’ supply, one Rx is assumed to be taken 30 days later. The start date of this Rx has 30 days added to it (ie, 14MAR21).	101 removed (0.6%) 8 modified start date (<0.1%)
**5. Opioid discontinuation** Prolonged gap of no opioid Rxs through end of study, corroborated by self-reported discontinuation of opioids on participant survey	In statistical analysis, set MME=0 for each study day after a participant’s last Rx. If uncorroborated, daily MME set to missing.	If a participant self-reported no longer taking opioids at the 12-month survey and the last Rx ended before the survey, daily MME was set to 0 for all study days after the last Rx end date.	22 subjects
**6. Suboxone for OUD** Suboxone Rx as treatment for opioid use disorder is a study exclusion criterion	In statistical analysis, set MME to missing for each study day starting at beginning of suboxone Rx.	If a participant was prescribed suboxone on day 440, MME was set to missing for each study day starting on day 440.	7 subjects

Quantity dispensed = total number of units to be taken over prescription duration; days’ supply = total number of days covered by the prescription duration; dose = total strength or quantity to be taken at one time; frequency = how many times to take the dose per day; Rx = prescription.

a Challenges are not mutually exclusive. A single prescription may be counted under multiple challenges. Percentages are calculated out of the 16 071 extracted opioid prescriptions relevant to the study’s design.

If at least one prescription within a set of matching medications with the same start date had an erroneous end date (Table 2, #1; Figure S2), and there was no subsequent gap indicative of a refill, it was assumed to be a faulty entry and was removed (2.9%). Another common pattern observed regularly within the same participants was overlapping prescriptions, defined as a set of 2 matching prescriptions where the start date of the second prescription was 1 or 2 days after the start date of the first prescription (Table 2, #2; Figure S3). The second prescription was removed, assuming it was an accidental duplicate (3.2%). Both assumptions were validated by the study’s subject matter expert (SME) and prescribing physicians via medication chart review.

Most opioids, and all potent opioids, are Schedule II medications (eg, hydromorphone, fentanyl), which cannot be refilled.^15^ For these opioids, clinicians may write sequential fills, or a series of two or three 30-day prescriptions written for the same start date, with the intent that prescriptions are filled at 30-day intervals. This was a recurring pattern across systems. When confirmed by a period of no prescriptions in the subsequent 30 or 60 days (Table 2, #3; Figure S4), the start dates were modified, assuming each prescription was filled in sequence (2.0%). Remaining sets of matching prescriptions with the same start date were considered spurious duplicates or early additional prescriptions (eg, those necessary when the initial prescription could only be partially filled) that could not be classified into one of the above categories. Physician review of these cases relative to the participants’ other opioid prescriptions identified 101 (0.6%) duplicates that were removed and 8 (<0.1%) that had 30 days added to the start date (Table 2, #4; Figure S5).

### Visualizing longitudinal opioid dosing for a patient

**Figure 3.**
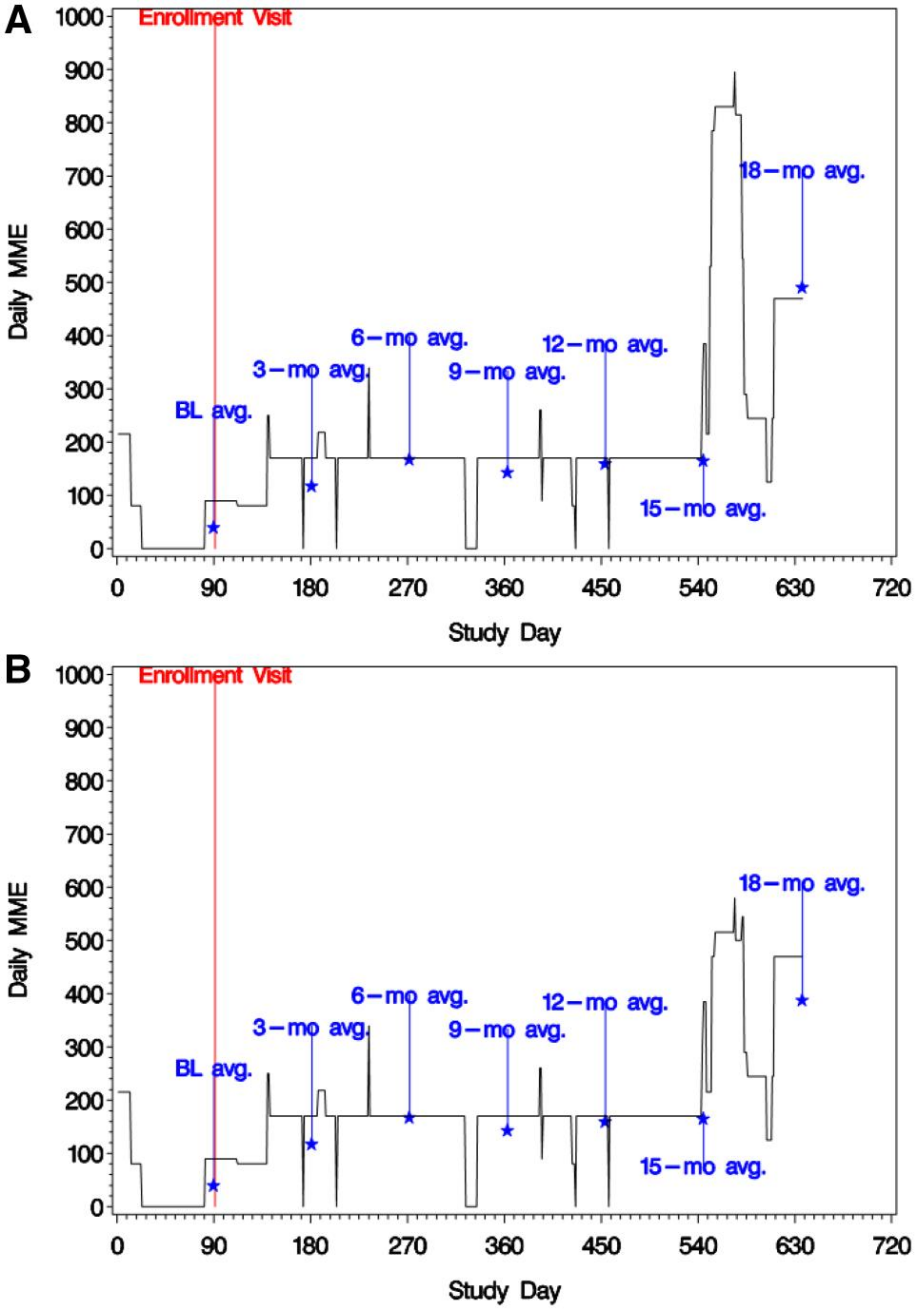
Example graphs of daily MME over the entire study period for a fictional participant, with average MME markers at the end of each 90-day period (where baseline is defined as day 1-90). Panel A depicts a spike between days 551 and 580, where there were three overlapping prescriptions for the same medication, assumed to be duplicates. Panel B shows the same participant’s graph after two of the duplicates were removed, bringing down the spike and the 18-month average by roughly 100 MME.

Individual-level graphs of daily MME were used to visualize longitudinal prescribing patterns to identify other potential data issues (Figure 3). For example, Panel A shows a spike in daily MME beginning on study day 551, where the participant was prescribed three 30-day prescriptions of oxycodone 5 mg. Through physician review of the patient’s other prescriptions, it was determined 2 were spurious duplicates and were removed (Table 2, #4), reducing the spike and dropping the 18-month average daily MME (Figure 3, Panel B).

### Opioid discontinuation and treatment for OUD

The final cleaning steps identified participants who permanently discontinued opioids during the study or were prescribed suboxone to treat an opioid use disorder (OUD) (an exclusion criterion). For the former, an MME of zero was assumed for each study day after a patient’s last opioid prescription ended, when corroborated by self-report on the study survey that they were no longer taking opioids (Table 2, #5; 4.2% or 22 subjects out of 525). Seven (1.3%) participants were prescribed suboxone to treat OUD after randomization; their daily MME were set to missing beginning on the first day of the prescription (Table 2, #6).

### Primary study analysis

In the final study analysis, a small decrease in average daily MME was observed from baseline to 12 months for both SDM (−6 MME; 95% CI, −14 to 2) and MI + CBT (−12 MME; 95% CI, −19 to −4). However, their mean difference was not statistically significant (*P* = .31).^16^

## Discussion

Although EHR prescription data were obtained using a standardized data model, many opioid prescriptions were missing information or contained discrepancies that required cleaning to derive daily MME. The most common prescription-level challenges were missing frequency or quantity dispensed. A potential solution that could be implemented within EHRs is to make certain fields required before a clinician can submit a prescription. Some discrepancies can be explained by differences in practice across sites. For instance, only one site excluded canceled prescriptions from their datamart. More complex issues were those involving imprecise start dates and apparent duplicate or overlapping entries, which may be attributable to providers’ prescribing patterns and individual patient circumstances. Study clinicians confirmed prescriptions that were likely spurious and should be removed from the derivation of longitudinal MME.

Limitations include basing MME on EHR prescriptions rather than those actually filled by a pharmacy, which would be more accurate. Additionally, lack of opioid prescriptions within a site’s EHR does not necessarily mean that the patient stopped taking them, as it is possible they changed healthcare systems. For these reasons, opioid researchers often use claims data from pharmacy records of filled prescriptions.^17^ However, claims data were not readily available for this clinical trial because patients use different insurers. Prescription drug monitoring programs (PDMPs) are state-level databases that track controlled substance prescriptions, making them an ideal source. However, they are highly restricted and their data are deidentified, resulting in limited use for research purposes.^18^ Further investigation is needed to compare accuracy of data between EHRs, PDMPs, and pharmacy prescription fills to determine the most efficient and reliable process for monitoring long-term opioid use. This is the first description of EHR opioid prescription data cleaning methods, so results cannot be compared against existing literature.

After completion of this study, the National Institutes of Health (NIH) released a new longitudinal MME calculator that considers multiple prescriptions over the same period and includes the conversion factors for all opioid classes.^19^ However, this tool does not address how to clean and prepare prescription data. This paper fills this gap to ensure that quality prescription data can be fed into such tools for accurate MME derivation.

Electronic opioid prescription data may be more accurate than patient self-report, though the literature comparing the two are limited. When research is focused on opioid tapering, self-report may be biased downward. Patients may also not be able to provide precise prescription details to accurately derive MME due to recall bias.^20^ An important strength of EHR prescriptions is that many systems require prescribers to enter information from lists of available options, maintaining quality. A standardized datamart supported by sites within a research network further simplifies the standardization process.

## Conclusion

In this study, EHR prescription data were used to standardize opioid doses and calculate longitudinal daily doses across participants. However, the programmers identified issues with prescription data that prevented MME derivation or had implausible dosing patterns, highlighting why prescription data cannot be taken at face value. An SME and prescribing physicians were consulted to develop a novel set of assumptions to systematically address these challenges. Other researchers who use EHRs for opioid prescription studies may use these steps to assure standardization and reduce risk of inaccuracies in prescribing patterns; however, these methods should not be considered definitive. Failure to clean and understand opioid prescription data invites a high risk of inaccurately evaluating longitudinal patterns and can potentially harm the management of patients’ health. Despite these challenges, electronic prescription data and MME estimations are valuable for both clinical practice and research.

## Supplementary Material

ooaf053_Supplementary_Data

## Data Availability

We plan to deposit data and data documentation in the Patient-Centered Outcomes Data Repository (PCODR) at the Inter-university Consortium for Political and Social Research (ICPSR).
