# Supplementary material for: Complexities and approaches for deriving longitudinal daily morphine milligram equivalents using electronic health record prescription data
Source: JAMIA Open. 2025 Jun 16;8(3):ooaf053. doi: 10.1093/jamiaopen/ooaf053 (PMC12169419; doi:10.1093/jamiaopen/ooaf053)
Supplement: ooaf053_Supplementary_Data [file ooaf053_supplementary_data.zip › Chang_EHR_Methods_Supplementary_Appendix.docx]

**SUPPLEMENTARY APPENDIX**

This supplementary material has been provided by the authors to give readers additional information about their work.

Supplement to: Chang, Hirsch, Thomas, et al. **Complexities and Approaches for Deriving Longitudinal Daily Morphine Milligram Equivalents (MME) using Electronic Health Record Prescription Data**

Table of Contents

[**Appendix Figure A1** 3](#_Toc172799984)

[**Appendix Table A1** 4](#_Toc172799985)

[**Appendix Table A2** 5](#_Toc172799986)

[**Appendix Figure A2** 6](#_Toc172799987)

[**Appendix Figure A3** 7](#_Toc172799988)

[**Appendix Figure A4** 8](#_Toc172799989)

[**Appendix Figure A5** 9](#_Toc172799990)

**Appendix Figure A1**. Diagram of data extraction from sites’ National Patient-Centered Clinical Research Network (PCORnet) Common Data Model (CDM) data warehouse.


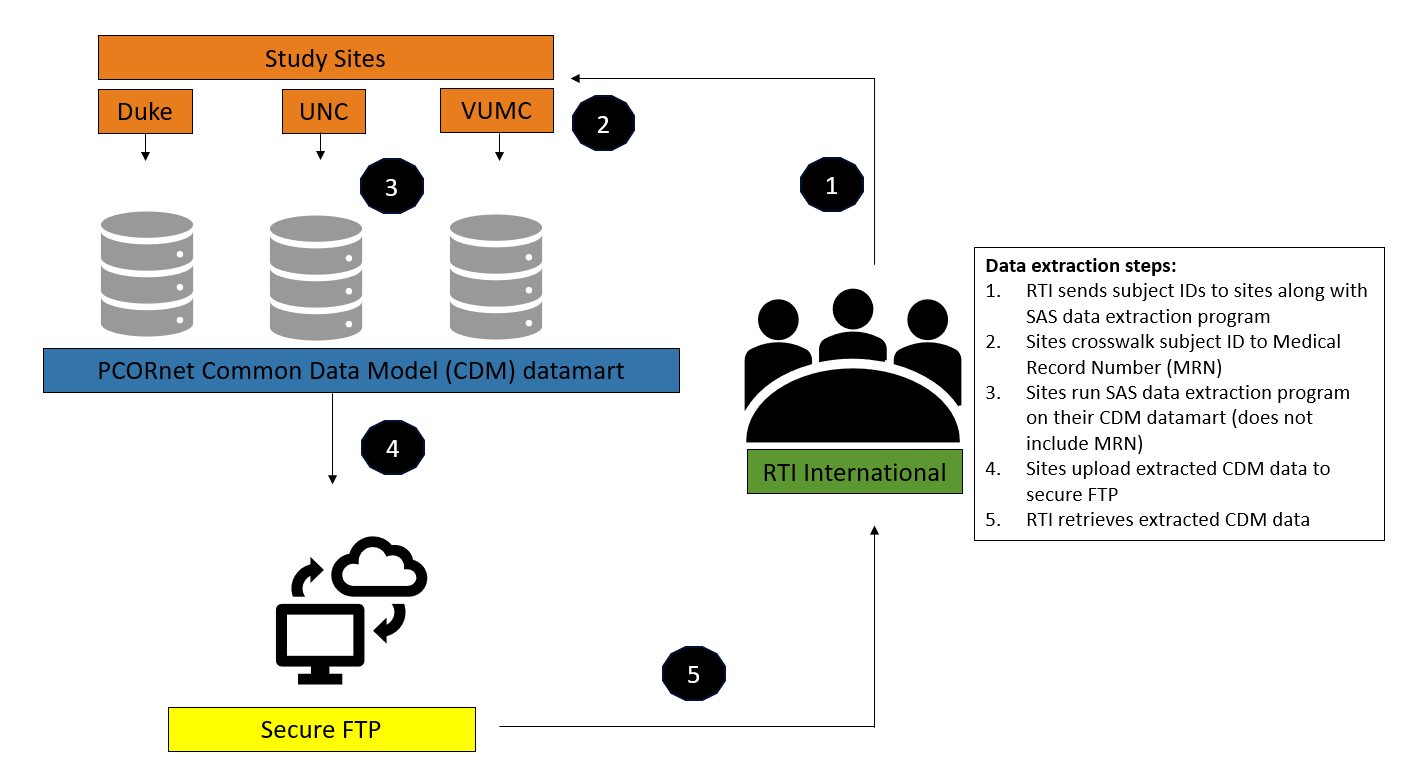


**Appendix Table A1.** Morphine milligram equivalent (MME) conversion factors by opioid type.

| **Opioid type^1^** | **MME conversion factor** |
| --- | --- |
| Buprenorphine^2^ |  |
| Transdermal patch (mcg/hour) | 12.6 per patch |
| Tablet and sublingual film (mg) | 30 |
| Buccal film (mcg) | 0.03 |
| Butorphanol (mg) | 7 |
| Codeine (mg) | 0.15 |
| Fentanyl, transdermal patch (mcg/hour) |  |
| 24-hour wear | 2.4 per patch |
| 48-hour wear | 4.8 per patch |
| 72-hour wear | 7.2 per patch |
| Hydrocodone (mg) | 1 |
| Hydromorphone (mg) | 4 |
| Levorphanol tartrate (mg) | 11 |
| Meperidine (mg) | 0.1 |
| Methadone (mg/day)^3^ |  |
| >0-20 | 4 per day |
| 21-40 | 8 per day |
| 41-60 | 10 per day |
| >60 | 12 per day |
| Morphine (mg) | 1 |
| Oxycodone (mg) | 1.5 |
| Oxymorphone (mg) | 3 |
| Tapentadol (mg) | 0.4 |
| Tramadol (mg) | 0.1 |

Note: There is no difference in conversion factors between long-acting and short-acting opioids.

^1^Unless otherwise noted, all conversion factors come from the CDC: <https://www.hhs.gov/guidance/sites/default/files/hhs-guidance-documents/Opioid%20Morphine%20EQ%20Conversion%20Factors%20%28vFeb%202018%29.pdf>

^2^The MME conversion factor for a buprenorphine patch is 1.8 for a 24-hour day. However, because buprenorphine patches are expected to be worn for 7 days, this conversion factor is multiplied by 7 (1.8 x 7= 12.6). Buprenorphine conversion factors come from: <https://www.ohiopmp.gov/Documents/MorphineEquivalentDailyDoseConversionTable.pdf>

^3^The conversion factor for methadone is not linear due to its long and unpredictable half-life. Methadone conversion factors come from the CDC: <https://www.cdc.gov/opioids/providers/prescribing/pdf/calculating-total-daily-dose.pdf>

**Appendix Table A2.** Frequencies of opioid type in final analytic set of prescriptions (N=14,864).

| **Opioid type** | **n (%)** |
| --- | --- |
| Oxycodone |  |
| Short-acting | 6,131 (41.2%) |
| Long-acting | 636 (4.3%) |
| Solution – SA | 13 (0.1%) |
| Hydrocodone SA |  |
| Tablet | 1,740 (11.7%) |
| Solution | 56 (0.4%) |
| Morphine |  |
| Long-acting | 1,109 (7.5%) |
| Short-acting | 505 (3.4%) |
| Solution – SA | 4 (<0.1%) |
| Fentanyl LA (patch) | 825 (5.6%) |
| Buprenorphine |  |
| Patch | 384 (2.6%) |
| Film | 232 (1.6%) |
| Tablet | 122 (0.8%) |
| Tramadol |  |
| Short-acting | 662 (4.5%) |
| Long-acting | 71 (0.5%) |
| Methadone |  |
| Tablet | 715 (4.8%) |
| Solution | 8 (0.1%) |
| Tapentadol | 700 (4.7%) |
| Hydromorphone |  |
| Short-acting | 558 (3.8%) |
| Long-acting | 66 (0.4%) |
| Solution – SA | 38 (0.3%) |
| Oxymorphone |  |
| Short-acting | 154 (1.0%) |
| Long-acting | 76 (0.5%) |
| Levorphanol | 32 (0.2%) |
| Butorphanol (nasal spray) | 11 (0.1%) |
| Codeine | 9 (0.1%) |
| Meperidine | 7 (0.1%) |

LA=long-acting; SA=short-acting

Note: Unless otherwise noted, medications are tablets or capsules.

**Appendix Figure A2.** Depiction of a set of duplicate prescriptions with the same start date (see **Table 2, #1**).

| 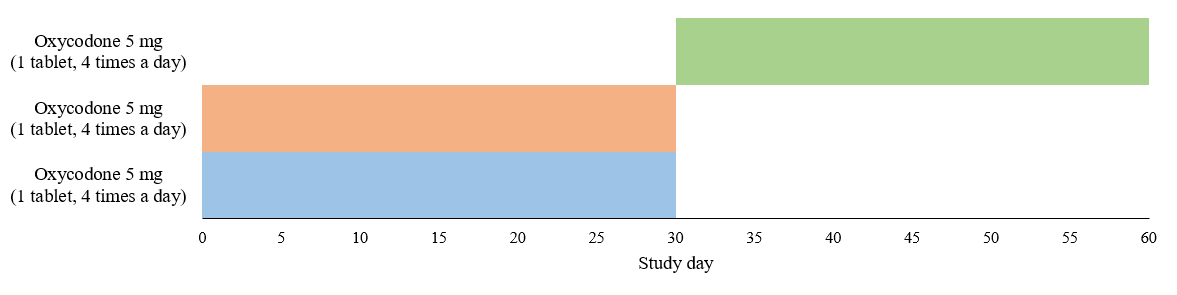  **PANEL A – Initial Prescriptions** |
| --- |
| 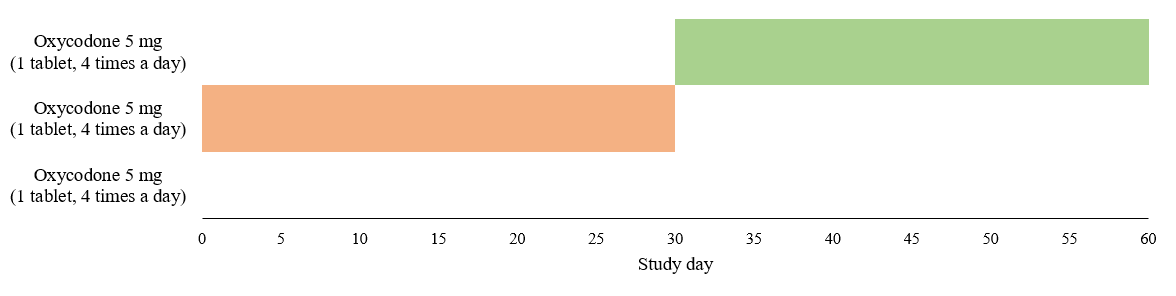  **PANEL B – Cleaned Prescriptions** |

[Panel A – Initial Prescriptions]: Two oxycodone 5 mg prescriptions written for the same start date, where one has a missing end date (not shown).

[Panel B – Cleaned Prescriptions]: The prescription with a missing end date is removed from the analytic dataset; prescriptions are assumed to be a pair of duplicates.

**Appendix Figure A3.** Depiction of a set of duplicate prescriptions, where one prescription begins two days after the first (see **Table 2, #2**).

| 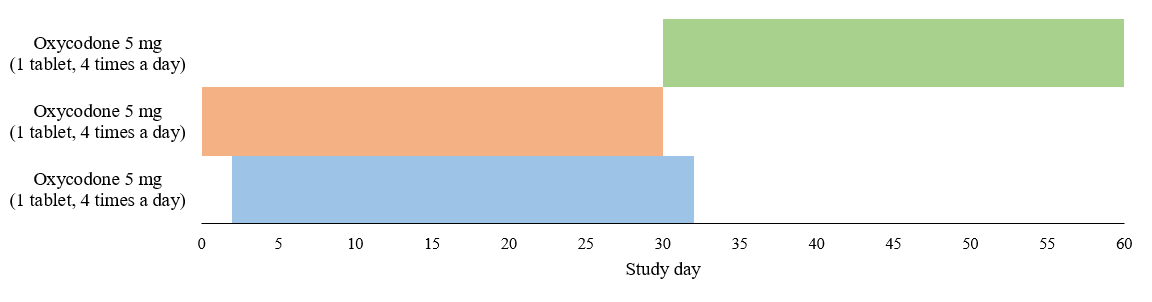  **PANEL A – Initial Prescriptions** |
| --- |
| 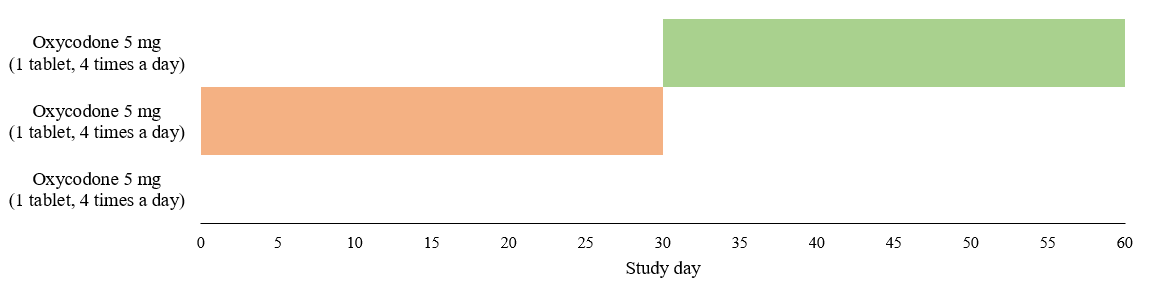  **PANEL B – Cleaned Prescriptions** |

[Panel A – Initial Prescriptions]: Two oxycodone 5 mg prescriptions with the same dosing instructions, quantity dispensed, and days’ supply, where the start date of the 2nd prescription is two days after the start date of the first prescription.

[Panel B – Cleaned Prescriptions]: The second prescription is assumed to be a duplicate and is removed from the analytic dataset.

**Appendix Figure A4.** Depiction of a series of three prescriptions assumed to be taken in sequence (see **Table 2, #3**).

| 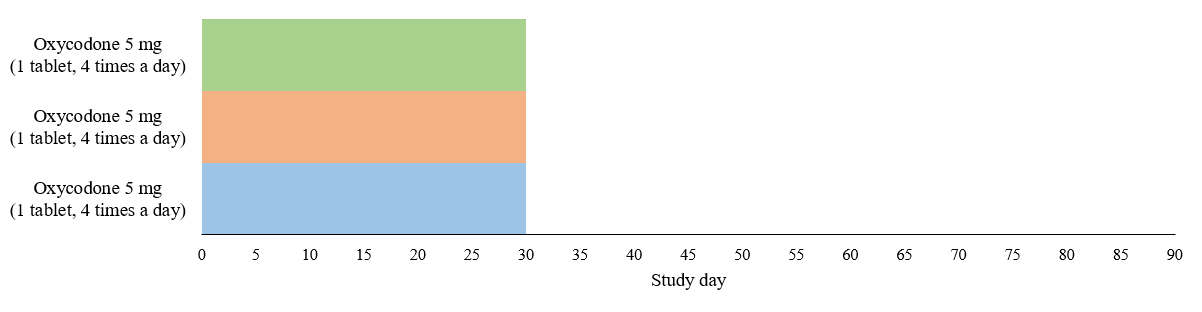  **PANEL A – Initial Prescriptions** |
| --- |
| 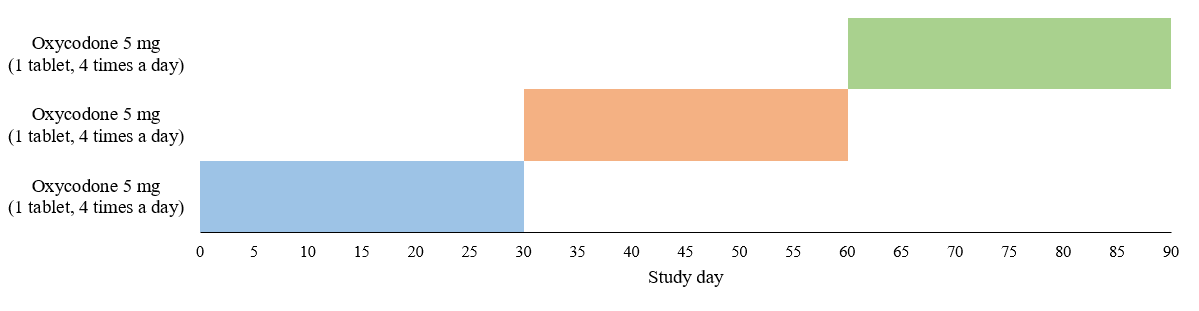  **PANEL B – Cleaned Prescriptions** |

[Panel A – Initial Prescriptions]: Three 30-day prescriptions of oxycodone 5 mg with the same start date.

[Panel B – Cleaned Prescriptions]: The three prescriptions were spread out over a total of 90 days since there were no other opioid prescriptions in the subsequent 60 days following their original end date; prescriptions were assumed to be taken in succession with no gaps.

**Appendix Figure A5.** Depiction of duplicates not recognized programmatically but identified during manual physician review (see **Table 2, #4**).

| 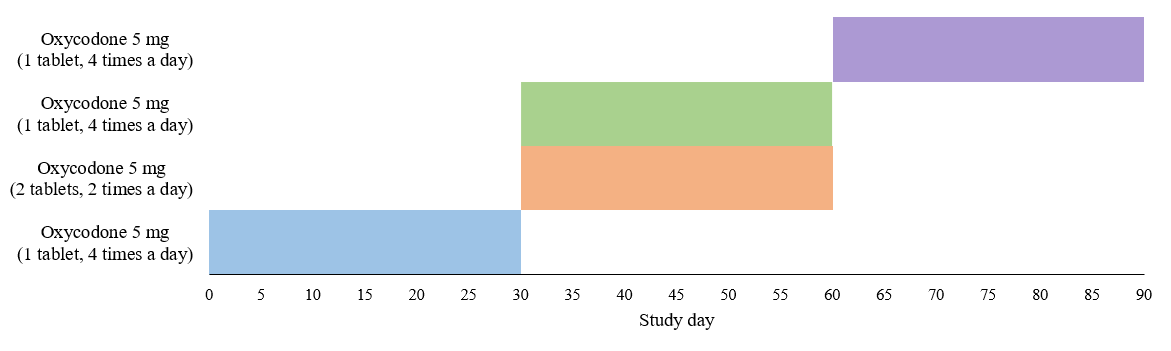  **PANEL A – Initial Prescriptions** |
| --- |
| 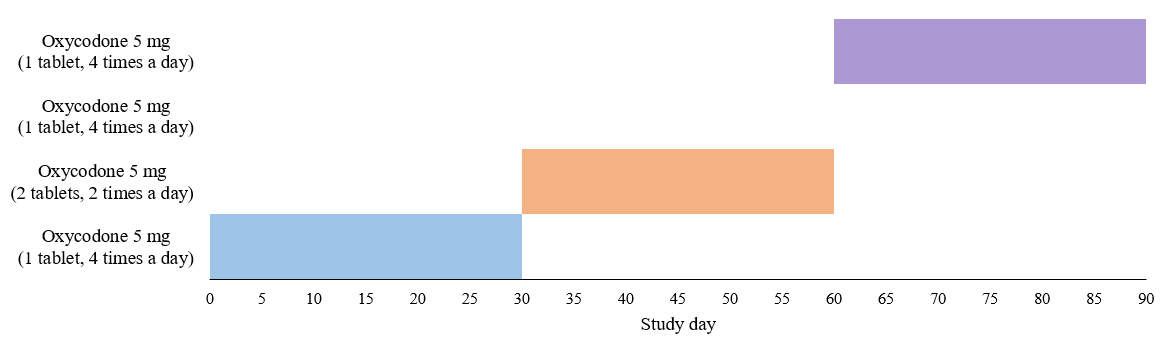  **PANEL B – Cleaned Prescriptions** |

[Panel A – Initial Prescriptions]: Two overlapping 30-day prescriptions of oxycodone 5 mg beginning on study day 30 (prescriptions were not diagnosed as duplicates programmatically because of their different dose ordered and frequency instructions).

[Panel B – Cleaned Prescriptions]: One of the oxycodone prescriptions beginning on study day 30 was removed from the analytic dataset after physician review determined it to be a duplicate.
