# Supplementary material for: Complexities and approaches for deriving longitudinal daily morphine milligram equivalents using electronic health record prescription data
Source: JAMIA Open. 2025 Jun 16;8(3):ooaf053. doi: 10.1093/jamiaopen/ooaf053 (PMC12169419; doi:10.1093/jamiaopen/ooaf053)
Supplement: ooaf053_Supplementary_Data [file ooaf053_supplementary_data.zip › Chang_EHR_Methods_AltText.docx]

**Figure 1 Alt text.** Example prescription, labeled A, indicating data components and formulas for calculating days’ supply, daily morphine milligram equivalents, total daily morphine milligram equivalents, and average morphine milligram equivalents per time period, labeled B to E.

**Figure 2 Alt text.** Flow diagram depicting steps taken in the study to remove opioid prescriptions that do not apply to study protocol, are missing data required to derive daily morphine milligram equivalents, or are spurious, with counts of how many were removed at each step.

**Figure 3 Alt text.** Graphs depicting daily morphine milligram equivalents for a single participant over the study period before and after duplicate prescriptions were removed from analysis, which resulted in a reduction of the 18-month average.

**Appendix Figure A1 Alt text.** Flow diagram depicting the steps of extracting electronic health records data from participating study sites, with each step labeled and described.

**Appendix Figure A2 Alt text.** Stacked horizontal bar charts illustrating opioid prescription coverages over a portion of study days before and after removing a duplicate prescription that starts on the same day as the matching prescription.

**Appendix Figure A3 Alt text.** Stacked horizontal bar charts illustrating opioid prescription coverages over a portion of study days before and after removing a duplicate prescription that starts two days after the matching prescription starts.

**Appendix Figure A4 Alt text.** Stacked horizontal bar charts illustrating opioid prescription coverages over a portion of study days before and after three overlapping prescriptions for the same medication are spread apart to start one immediately after another.

**Appendix Figure A5 Alt text.** Stacked horizontal bar charts illustrating opioid prescription coverages over a portion of study days before and after removing a duplicate prescription that was identified manually.
